# Resolution–noise characteristics of common FDK filter kernels: A practical reference for preclinical cone-beam micro-CT

**DOI:** 10.1371/journal.pone.0349360

**Published:** 2026-08-18

**Authors:** Falk L. Wiegmann, Nancy L. Ford

**Affiliations:** 1 Department of Physics and Astronomy, The University of British Columbia, Vancouver, British Columbia, Canada; 2 Department of Oral Biological and Medical Sciences, The University of British Columbia, Vancouver, British Columbia, Canada; Medical Center - University of Freiburg, GERMANY

## Abstract

The ramp filter kernel and cutoff frequency are fundamental parameters of the Feldkamp–Davis–Kress (FDK) algorithm that determine the resolution and noise characteristics of the reconstructed image. Despite their importance, the filter configuration is frequently unreported in preclinical micro-CT studies. We reconstruct identical data from a GE eXplore CT 120 scanner using four filter kernels (ramp, Shepp-Logan, cosine, Hamming) at four cutoff frequencies (1.0, 0.8, 0.6, and 0.379× Nyquist, matched to the detector-to-voxel size ratio) and evaluate each of the sixteen configurations using the modulation transfer function (MTF), noise power spectrum (NPS), and non-prewhitening detectability index (NPW $d^{\prime }$). Qualitative assessment is performed on a mouse lung specimen. Across the sixteen configurations, MTF_10_ ranges from 1.74 to 3.22 lp/mm, integrated NPS from 75 670 to 13 259 HU^2^, and the Rose criterion crossing diameter from 2.03 to 0.90 mm at ΔC=500 HU and from 6.89 to 3.56 mm at 100 HU. This note presents the data as a concise, primarily comparative visual and quantitative reference for FDK filter selection in preclinical cone-beam CT.

## Introduction

The Feldkamp–Davis–Kress (FDK) algorithm [1] is the standard reconstruction method for cone-beam micro-CT [2]. A central step in the FDK pipeline is ramp filtering: each detector row is convolved with a filter whose ideal frequency response is |*f*|, compensating for the non-uniform sampling density inherent in the projection-slice geometry [3,4].

In practice, the ideal ramp is never used directly because its unbounded gain at high frequencies amplifies noise. Instead, the ramp is multiplied by an apodization window *W*(*f*) that attenuates high-frequency content, and the result is truncated at a cutoff frequency $f_{c}$. Four classical windows are in common use: none (the pure ramp, also known as Ram-Lak [3]), Shepp-Logan [5], cosine, and Hamming [4]. Together, the window and cutoff determine the resolution and noise characteristics of the reconstructed image.

Despite the importance of these parameters, most preclinical micro-CT studies either do not report their filter configuration or select a single setting without systematic justification. This note provides a reference for filter selection by sweeping four windows across four cutoff frequencies on identical data and evaluating each configuration using the modulation transfer function (MTF), noise power spectrum (NPS), and non-prewhitening (NPW) detectability index $d^{\prime }$ [6], a task-based figure of merit that combines signal transfer (MTF) and noise (NPS) into a single measure of how well a specified object can be detected. Filter and cutoff effects on cone-beam CT image quality have been examined previously [7–14], but typically with a single image-quality metric or a single filter family in isolation. This note evaluates MTF, NPS, and task-based detectability on identical scan data across the full window–cutoff grid, including the physically motivated matched cutoff $f_{c}=d_{a}/\Delta x$, and presents them as a single visual reference.

### Background

The FDK ramp filter in the frequency domain takes the form


$$\begin{array}{c} H(f)=|f|\cdot W(f),\;|f|\leq f_{c}, \end{array}$$ (1)

where $f_{c}$ is the cutoff frequency and *W*(*f*) is the apodization window. The four windows evaluated in this study are:


$$\begin{array}{c} \begin{array}{cc} \text{Ramp (Ram-Lak):}\; & W(f)=1, \end{array} \end{array}$$ (2)

$$\begin{array}{c} \begin{array}{cc} \text{Shepp-Logan:}\; & W(f)=sinc\;\left(\frac{f}{2f_{c}}\right), \end{array} \end{array}$$ (3)

$$\begin{array}{c} \begin{array}{cc} \text{Cosine:}\; & W(f)=\cos\;\left(\frac{\pi f}{2f_{c}}\right), \end{array} \end{array}$$ (4)

$$\begin{array}{c} \begin{array}{cc} \text{Hamming:}\; & W(f)=0.54+0.46\cos\;\left(\frac{\pi f}{f_{c}}\right). \end{array} \end{array}$$ (5)

These windows provide progressively stronger high-frequency attenuation, from no apodization (ramp) to substantial suppression (Hamming). All are zero for $|f|>f_{c}$.

The cutoff frequency $f_{c}$ is expressed as a fraction of the detector Nyquist frequency $f_{det}=1/(2d_{a})$, where $d_{a}$ is the detector pixel pitch. For the eXplore CT 120 scanner ($d_{a}=0.0284\;mm$), $f_{det}=17.6\;lp/mm$. A physically motivated choice is the *matched* cutoff $f_{c}=(d_{a}/\Delta x)\cdot f_{det}=1/(2\Delta x)$, where Δx is the reconstruction voxel size. We adopt Δx=0.075 mm as our reference voxel spacing, for which $d_{a}/\Delta x=0.379$ and $f_{c}=6.67\;lp/mm$—exactly the reconstruction Nyquist frequency, beyond which the voxel grid cannot represent additional detail.

## Methods

### Scanner and acquisition

All data were acquired on the GE eXplore CT 120 micro-CT scanner at 80 kVp, 40 mA, with 16 ms exposure per frame and no frame averaging. The flat-panel detector comprises 3500×2300 pixels with a pixel pitch of 0.0284 mm. Table 1 summarises the scanner geometry. A short-scan acquisition (193°, 220 projections) was used for all reconstructions.

**Table 1:** Scanner geometry parameters for the eXplore CT 120.

Parameter	Value
Detector size (pixels)	3500×2300
Detector pixel pitch (mm)	0.0284
Source-to-detector distance (mm)	451.5
Source-to-isocentre distance (mm)	396.4
Scan arc	193° ($180^{\circ }+2\gamma_{m}$)
Number of projections	220

### Specimens

Quantitative image quality was evaluated on the mCTP 610 image quality phantom (Shelley Medical Imaging Technologies, Canada), which contains a slanted-edge insert (air/acrylic boundary) for MTF measurement and a homogeneous water region for NPS characterisation. Visual comparison was performed on a mouse lung specimen acquired with respiratory gating as part of a previously published study [15].

### Ethics statement

No animal experiments were conducted as part of this study. The image quality phantom data were acquired exclusively for evaluation purposes. The *in vivo* mouse micro-CT data were collected in prior studies under protocols approved by the University of British Columbia Animal Care Committee and performed in accordance with the ARRIVE guidelines [16]: a thoracic FLASH radiotherapy study (Protocol No. A21-0060, approved August 31, 2021) [15,17], and a baseline imaging study (Protocol No. A24-0150), the latter previously unpublished. The imaging data were re-used here for reconstruction evaluation only. No animals were imaged, handled, or subjected to any procedures as part of this work.

### Reconstruction

Reconstructions were performed using our open-source FDK pipeline [2] on a 1247×1247×933 grid with isotropic 0.075 mm voxels. The pipeline processes each projection p(β,u,v) at gantry angle β with detector coordinates (*u*, *v*) through the following steps:

1. **Pre-processing**: dark-current subtraction, flood-field normalisation, and logarithmic transform to convert raw detector intensities to line integrals of attenuation.
2. **Cosine weighting**: multiplication by $D/\sqrt{D^{2}+u^{2}+v^{2}}$, where *D* is the source-to-detector distance, to account for varying path lengths in cone-beam geometry.
3. **Parker weighting**: for short-scan acquisitions, multiplication by smooth weighting functions that correct for non-uniform data redundancy near the scan-arc boundaries [18,19].
4. **Ramp filtering**: one-dimensional convolution of each detector row with a filter kernel H(f)=|f|·W(f) as defined in Eq (1), with window *W*(*f*) and cutoff $f_{c}$ as described in the Background section.
5. **Backprojection**: three-dimensional voxel-driven backprojection with distance weighting.
6. **HU calibration**: conversion from linear attenuation to Hounsfield units using air and water reference values.

**Fig 1:**
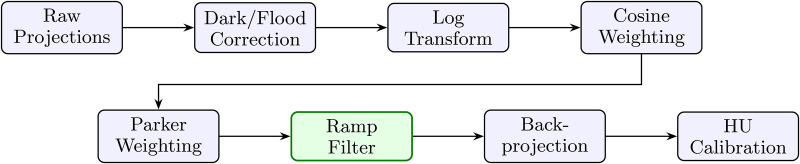
FDK reconstruction pipeline for short-scan cone-beam micro-CT. The ramp filter step (highlighted) is the only step varied in this study.

Fig 1 summarises this pipeline. Step 4 (ramp filtering) is the only step varied in this study; sixteen configurations were evaluated by combining four filter kernels (ramp, Shepp-Logan, cosine, Hamming) with four cutoff frequencies (1.0, 0.8, 0.6, and 0.379× Nyquist).

### Image quality metrics

#### Modulation transfer function.

The MTF was measured from the slanted-edge insert using an ISO 12233-style [20] oversampled edge technique with 4× subpixel alignment. The line spread function was obtained by direct differentiation of the oversampled edge response function, and the MTF was computed as the normalised magnitude of its Fourier transform. Spatial resolution is reported as the frequencies at which the MTF falls to 50% and 10% of its peak value (MTF_50_ and MTF_10_). Uncertainty was estimated by a 500-sample bootstrap over the edge slices and is shown as shaded bands.

#### Noise power spectrum.

The NPS was measured following ICRU Report 87 [21] from eight circular ROIs (radius = 66 pixels) arranged in a ring pattern (0° to 315° in 45° steps) over 16 axial slices in the homogeneous water region of the phantom. A third-order polynomial was subtracted from each ROI to remove low-frequency trends. The 2D NPS was computed as $|FFT{|}^{2}$, normalised by $\Delta x^{2}/(\pi r^{2}N_{ROI})$, and radially averaged. The integrated NPS was obtained by 2D Simpson integration. Uncertainty is the standard deviation across the eight ROIs.

#### Detectability index.

Task-based detectability was quantified using the non-prewhitening matched filter (NPW) observer model [6]. The task function was a circular disc of contrast ΔC and radius *R*: $T(f)=\Delta C\;\pi R^{2}\;2J_{1}(2\pi Rf)/(2\pi Rf)$. The detectability index was computed as


$$\begin{array}{c} d^{\prime 2}=\frac{{\left[\int_{0}^{f_{max}}|T(f){|}^{2}\;{MTF}^{2}(f)\;f\;df\right]}^{2}}{\int_{0}^{f_{max}}|T(f){|}^{2}\;{MTF}^{2}(f)\;NPS(f)\;f\;df} \end{array}$$ (6)

at two contrast levels: ΔC=500 HU (high-contrast tasks, e.g., iodine-enhanced vessels or bone–soft-tissue boundaries) and ΔC=100 HU (unenhanced soft-tissue lesion detection). Disc diameters spanned 0.1–3.5 mm at 500 HU and 0.1–9 mm at 100 HU, ensuring every configuration crosses the Rose threshold. The Rose criterion [22] ($d^{\prime }=3$) was used to define the minimum detectable disc diameter for each configuration at each contrast level. Uncertainty in $d^{\prime }$ and the Rose crossing was propagated from the MTF and NPS bootstraps.

### Use of generative AI tools

Claude (Anthropic) was used to assist with manuscript preparation. It was also used to assist with figure generation from acquired results and code repository maintenance and cleanup. All AI-generated content was reviewed, verified, and revised by the authors, who take full responsibility for the final manuscript.

## Results

### MTF, NPS, and detectability

**Fig 2:**
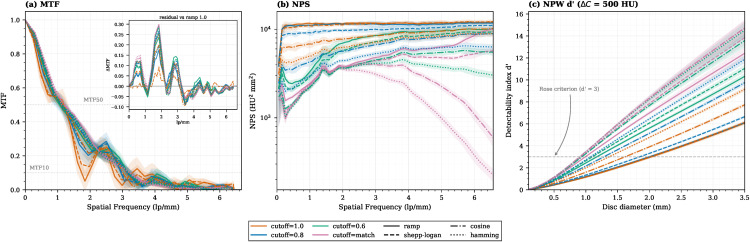
Image quality metrics for all sixteen filter configurations on the image quality phantom (short-scan, 193°). (a) MTF, with the inset showing the residual relative to the ramp at $f_{c}=1.0$; (b) NPS; (c) NPW detectability index at ΔC=500 HU, with the dashed line marking the Rose criterion ($d^{\prime }=3$). Line style denotes filter kernel (solid ramp, dashed Shepp-Logan, dash-dot cosine, dotted Hamming) and colour denotes cutoff; shaded bands are ±1 standard deviation. Higher cutoff and weaker apodization raise noise (b) with little change in resolution (a).

Fig 2 presents the MTF, NPS, and NPW $d^{\prime }$ for all sixteen filter configurations on the image quality phantom.

The MTF curves (panel a) overlap closely across all sixteen configurations up to the MTF_50_ level (MTF${\;}_{50}\approx 1.1$–1.2 lp/mm), indicating that the measured spatial resolution is essentially system-limited rather than filter-limited in this regime. The inset shows the residual MTF of each configuration relative to the ramp at $f_{c}=1.0$. Table 2 lists the MTF_10_ values for all sixteen configurations.

**Table 2:** MTF_10_ (lp/mm) for each filter kernel and cutoff frequency. Values are mean ± standard deviation; best value (highest MTF_10_) in bold.

Cutoff	Ramp	Shepp-Logan	Cosine	Hamming
1.0	1.74 ± 0.73	2.84 ± 0.67	3.01 ± 0.21	3.12 ± 0.16
0.8	2.95 ± 0.47	2.98 ± 0.31	3.14 ± 0.15	3.16 ± 0.14
0.6	3.16 ± 0.17	3.17 ± 0.17	3.19 ± 0.15	3.20 ± 0.14
Match	3.21 ± 0.20	3.20 ± 0.18	**3.22 ± 0.13**	3.20 ± 0.10

The NPS (panel b) shows variation in both the magnitude and spectral shape of image noise across configurations. Configurations with higher cutoffs show elevated high-frequency noise power. Table 3 lists the integrated NPS for each configuration.

**Table 3:** Integrated NPS (HU^2^) for each filter kernel and cutoff frequency. Values are mean ± standard deviation; best value (lowest integrated NPS) in bold.

Cutoff	Ramp	Shepp-Logan	Cosine	Hamming
1.0	75 670 ± 2695	74 633 ± 3580	55 174 ± 3677	46 160 ± 3030
0.8	73 795 ± 3800	64 970 ± 4049	43 543 ± 2883	32 781 ± 2019
0.6	49 783 ± 3426	44 113 ± 2950	27 895 ± 1565	22 105 ± 1184
Match	33 354 ± 1529	26 458 ± 1266	17 742 ± 946	**13 259 ± 713**

The NPW $d^{\prime }$ (panel c) combines both effects into a single task-relevant metric. Table 4 lists the Rose criterion [22] crossing diameters at ΔC=500 HU; Table 5 lists the corresponding crossings at ΔC=100 HU, a lower-contrast regime in which detectability requires substantially larger lesion diameters across all configurations.

**Table 4:** Rose criterion crossing diameter (mm) at ΔC=500 HU. The smallest disc diameter for which $d^{\prime }\geq 3$; smaller values indicate better detectability. Values are mean ± standard deviation; best (smallest) values in bold.

Cutoff	Ramp	Shepp-Logan	Cosine	Hamming
1.0	2.03 ± 0.06	2.01 ± 0.05	1.62 ± 0.03	1.36 ± 0.02
0.8	2.00 ± 0.04	1.85 ± 0.03	1.28 ± 0.02	1.07 ± 0.01
0.6	1.17 ± 0.03	1.10 ± 0.02	**0.90 ± 0.01**	**0.90 ± 0.01**
Match	0.99 ± 0.01	0.92 ± 0.01	**0.90 ± 0.01**	**0.90 ± 0.01**

**Table 5:** Rose criterion crossing diameter (mm) at ΔC=100 HU. The low-contrast counterpart to Table 4; smaller values indicate better detectability. Values are mean ± standard deviation; best (smallest) value in bold.

Cutoff	Ramp	Shepp-Logan	Cosine	Hamming
1.0	6.82 ± 0.08	6.82 ± 0.09	5.91 ± 0.11	5.29 ± 0.12
0.8	6.89 ± 0.10	6.53 ± 0.10	5.05 ± 0.13	4.33 ± 0.15
0.6	4.83 ± 0.13	4.60 ± 0.14	3.86 ± 0.17	3.61 ± 0.17
Match	4.19 ± 0.19	3.79 ± 0.18	**3.56 ± 0.17**	3.57 ± 0.17

### Spatial resolution vs cutoff

**Fig 3:**
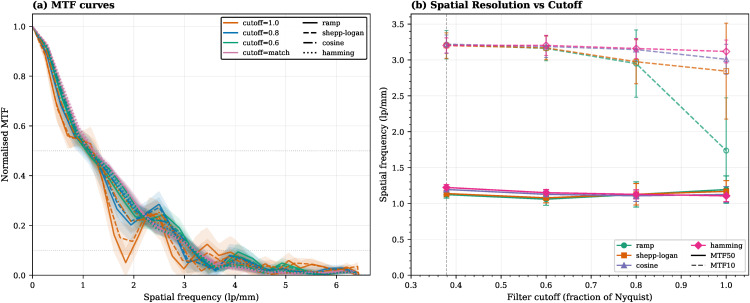
Spatial resolution as a function of filter configuration. (a) MTF curves for the sixteen configurations, with the MTF_50_ and MTF_10_ levels marked. (b) MTF_50_ (solid) and MTF_10_ (dashed) versus cutoff for each kernel; the vertical dashed line marks the matched cutoff $d_{a}/\Delta x=0.379$. Shaded bands and error bars are ±1 standard deviation. The curves overlap up to MTF_50_; the wider MTF_10_ spread at high cutoff is noise-driven.

Fig 3 provides a complementary view of how spatial resolution varies with filter configuration. Panel (a) shows the full MTF curves for all sixteen configurations. Panel (b) plots MTF_50_ and MTF_10_ as a function of cutoff frequency for each filter kernel, showing the continuous relationship between cutoff and measured spatial resolution. The matched cutoff $d_{a}/\Delta x=0.379$ is marked for reference.

### Qualitative comparison on mouse images

**Fig 4:**
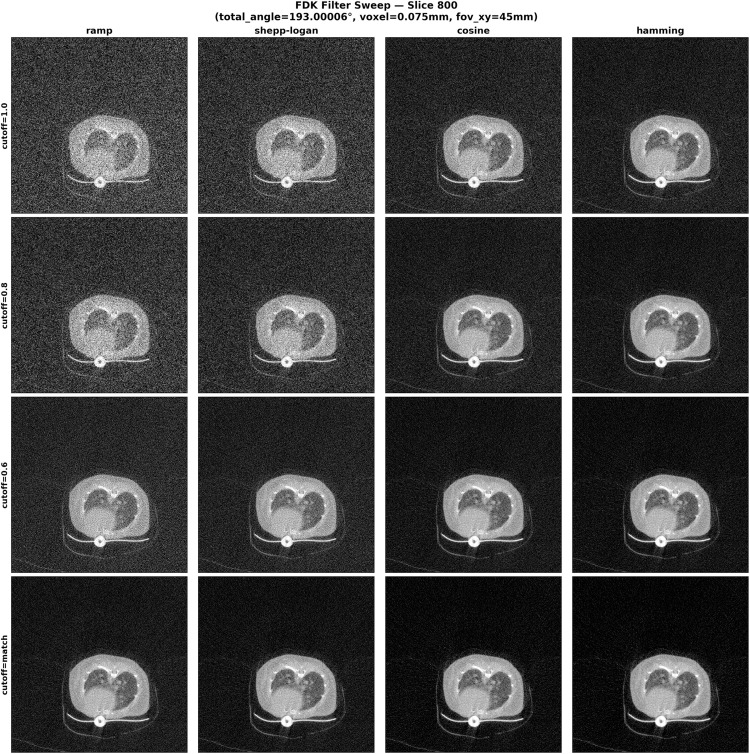
Mouse lung reconstructions (axial slice) for all sixteen filter configurations, displayed with identical window/level settings. Columns: ramp, Shepp-Logan, cosine, Hamming; rows: $f_{c}=1.0$, 0.8, 0.6, matched (0.379× Nyquist). Noise is coarsest at top-left (high cutoff, weak apodization) and smoothest at bottom-right (matched cutoff, Hamming).

Fig 4 presents axial slices of a mouse lung reconstructed with each of the sixteen configurations. The grid spans filter kernels (columns) and cutoff frequencies (rows), with all images displayed at identical window and level settings. The visual differences in noise texture and structural detail across configurations complement the quantitative metrics presented above.

**Fig 5:**
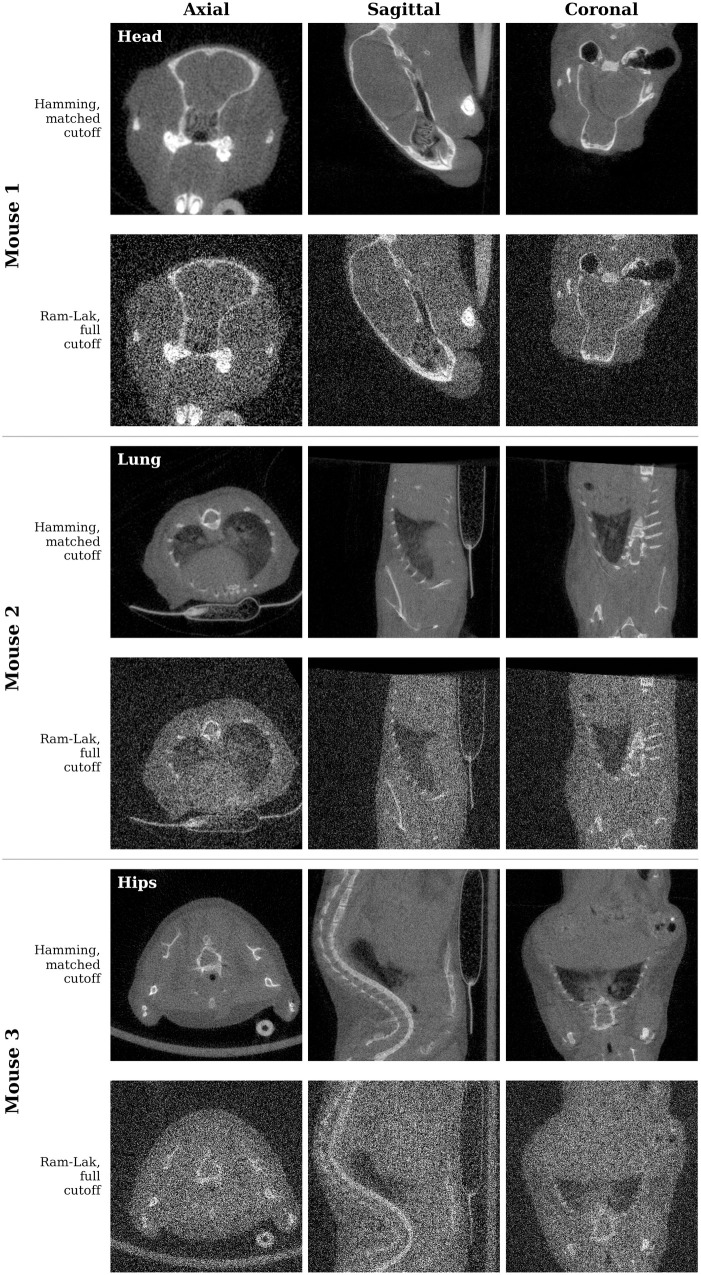
Best versus worst filter configuration across mice, anatomy, and imaging planes. The best (Hamming, matched cutoff) and worst (ramp, full cutoff) configurations, identified from the phantom image-quality metrics, applied to three mice in axial, sagittal, and coronal planes (columns). Within each mouse, the top row is the best configuration and the bottom row the worst. Mouse 1 (head) and Mouse 2 (lung) are untreated female mice aged 12 weeks (unpublished data). Mouse 3 (hips) is a male mouse aged 22–24 weeks, imaged outside the irradiated field of a prior thoracic FLASH radiotherapy study [15]. All images use identical window and level settings. The noise reduction of the recommended configuration is consistent across subjects, anatomical regions, and planes.

To assess whether the filter-induced differences observed on the phantom translate to varied biological subjects, Fig 5 compares the best (Hamming, matched cutoff) and worst (ramp, full cutoff) configurations, identified from the phantom metrics, across three mice and three anatomical regions, each in three orthogonal planes. The noise reduction afforded by the recommended configuration is evident across all subjects, regions, and planes, indicating that the qualitative benefit is not specific to a single specimen or anatomical site.

## Discussion

The data presented here show that filter kernel and cutoff frequency selection substantially affects image noise and task-based detectability, while having little effect on the resolution-determining part of the MTF. Because every cutoff studied lies above the system resolution limit (MTF${\;}_{50}\approx 1.1$–1.2 lp/mm, set by detector and focal-spot blur), the filter choice primarily reshapes the high-frequency noise rather than the spatial resolution; MTF_50_ is therefore essentially constant across the sixteen configurations, and the apparent variation in MTF_10_ is driven by noise in the MTF tail rather than by a true change in resolution. The NPS data in particular illustrate that the spectral distribution of noise, not just its magnitude, varies with filter configuration: configurations with higher cutoffs and weaker apodization concentrate more noise power at higher spatial frequencies, while stronger apodization reshapes the noise spectrum toward a flatter profile. The NPW $d^{\prime }$ captures how these spectral characteristics combine for a given detection task, providing a single metric that accounts for both signal transfer and noise. The detectability index assumes an idealised circular-disc task with a non-prewhitening observer. Real preclinical imaging tasks may differ substantially in morphology and contrast, so the Rose-criterion diameters are best read as comparative benchmarks rather than predictions for any specific task.

The matched cutoff $f_{c}=d_{a}/\Delta x$ provides a physically motivated default by limiting the filter bandwidth to frequencies that are meaningfully sampled given the detector pixel pitch and reconstruction voxel size. This is the configuration used in our companion benchmarking study [2], where the Hamming kernel at matched cutoff was adopted for the FDK pipeline.

These results are specific to the eXplore CT 120 platform and phantom: the absolute resolution and noise values also depend on system-specific factors and acquisition settings beyond the reconstruction filter, such as detector response, focal-spot blur, magnification, interpolation, reconstruction implementation, dose, and projection count. The matched cutoff also differs for other detector pitches or voxel sizes. They should therefore be read as a comparative characterisation of the filter-induced resolution–noise trade-off rather than as absolute values transferable to other systems, imaging tasks, or specimens. Beyond the phantom, the qualitative comparison in Fig 5 shows that the noise benefit of the recommended configuration persists across multiple mice that differ in sex, age, and treatment history, and across head, lung, and pelvic anatomy in three orthogonal planes, indicating that the comparative trends are not specific to a single specimen or site. The biological images remain illustrative rather than a basis for quantitative claims, since the quantitative metrics were measured on the phantom.

## Conclusion

This note provides a systematic visual and quantitative reference for FDK filter kernel and cutoff frequency selection in preclinical cone-beam micro-CT. The sixteen configurations evaluated span a wide range of image quality characteristics, and the data are presented to assist groups in selecting filter parameters appropriate to their imaging task; as the absolute values are platform-specific, it is the comparison between configurations that transfers to other systems.
